# Evaluation of awareness & utilisation of clinical practise guideline for management of adult Dengue infection among Malaysia doctors

**DOI:** 10.1371/journal.pone.0178137

**Published:** 2017-05-31

**Authors:** Mohd Izhar Ariff, Abqariyah Yahya, Rafdzah Zaki, Roza Sarimin, Izzuna Mudla Mohamed Ghazali, Balvinder Singh Gill, Zailiza Suli, Mohd. Aminuddin Mohd. Yusof, Nafisah Ahmad Lutfi, Sin Lian Thye, Fatanah Ismail, Maimunah Mahmud, Rugayah Bakri

**Affiliations:** 1 Department of Social & Preventive Medicine, Faculty of Medicine, University of Malaya, Kuala Lumpur, Malaysia; 2 Julius Centre University Malaya, Department of Social & Preventive Medicine, Faculty of Medicine, University of Malaya, Kuala Lumpur, Malaysia; 3 Health Technology Assessment Section, Medical Development Division, Ministry of Health, Putrajaya, Malaysia; 4 Information and Documentation Surveillance section, Disease Control Division, Ministry of Health, Putrajaya, Malaysia; 5 Family Health Division, Ministry of Health, Putrajaya, Malaysia; 6 Institute for Health Management, Ministry of Health, Putrajaya, Malaysia; 7 Jinjang Health Clinic, Ministry of Health, Putrajaya, Malaysia; 8 Health Technology Assessment Section, Medical Development Division, Ministry of Health, Putrajaya, Malaysia; Institute of Tropical Medicine (NEKKEN), Nagasaki University, JAPAN

## Abstract

Clinical Practice Guideline (CPG) provides evidence-based guidance for the management of Dengue Infection in adult patients. A cross sectional study was conducted to evaluate awareness and utilization of CPG among doctors in public or private hospitals and clinics in Malaysia. Doctors practicing only at hospital Medical and Emergency Departments were included, while private specialist clinics were excluded in this study. A multistage proportionate random sampling according to region (Central, Northern, Southern, Eastern, Sabah and Sarawak) was performed to select study participants. The overall response rate was 74% (84% for public hospitals, 82% for private hospitals, 70% for public clinics, and 64% for private clinics). The CPG Awareness and Utilization Feedback Form were used to determine the percentage in the study. The total numbers of respondent were 634 with response rate of 74%. The mean lengths of service of the respondent were 13.98 (11.55).A higher percentages of doctors from public facilities (99%) were aware of the CPG compared to those in private facilities (84%). The percentage of doctors utilising the CPG were also higher (98%) in public facilities compared to private facilities (86%). The percentage of Medical Officer in private facilities that utilizing the CPG were 84% compares to Medical Officer in public facilities 98%. The high percentage of doctors using the CPG in both public (97%) and private (94%) hospitals were also observed. However, only 69% of doctors in private clinics utilised the CPG compared to doctors in public clinics (98%). Doctors in both public and private facilities were aware of the dengue CPG. However, most doctors in private clinic were less likely to utilise the CPG. Therefore, there is a need to increase the level of CPG utilisation especially in private clinics.

## Introduction

Dengue is currently an important public health problem globally with significant socioeconomic and disease burden [1]. The World Health Organisation (WHO) estimated that 50–100 million dengue infections occur worldwide every year [2]. Almost half a million of patients with dengue haemorrhagic fever is hospitalized and many of them are children with 2.5 per cent of these patients die [2].

Dengue has been classified as the most important mosquito-borne viral disease in the world due to significant geographic spread of the virus and the subsequent costly burden of disease it brings [3]. Dengue infection poses major challenge to public health with its worldwide spread and dramatic increase in incidence in the tropical regions of Latin America, the Caribbean and Asia [4, 5]. Asia remains disproportionately affected with 75% of the global disease burden borne by the populations within the Southeast Asia and Western Pacific regions [6, 7].

In Malaysia, the Vector Borne Disease Control Programme conducts dengue control activities based on case surveillance through case notification. Vector control activities are carried out by vector surveillance via regular entomological surveys, source reduction and space spraying as well as public participation through Communication for Behavioural Impact (COMBI) programme [8, 9]. The case fatality rate (CFR) of dengue cases has been a major issue since before as in 2003 the number of dengue death is 72 then the dengue death rate keep increasing to 134 dengue deaths in 2010. After that the number of dengue death in Malaysia when down in 2011 and 2012 with 35 to 36 dengue death per year [10]. Despite, Malaysia is currently experiencing a surge of dengue cases with 108,698 cases reported in 2014, which is 151% increases compared to 43,346 cases in 2013[11]. There has also been an increase in dengue mortality with 215 deaths reported in 2014 compared to 92 in 2013 [11]. At present, there is one dengue vaccine develop BY Sanofi Pasteur has been licensed which is Dengvaxia (CYD-TDV). Two Phase 3 clinical trials of CYD-TDV were done, CYD14 in Asia and CYD15 in Latin America, where more than 35,000 participants were included from aged 2 to 16 years. The result of the vaccine efficacy varied by serotypes and the efficacy during the initial time was 79.1%. Also, the efficacy varied by age at vaccination where the efficacy was 65.6% for participants aged more than 9 years old and it was 44% in participants below 9 years old. The recommendations of WHO is introduction of CYD-TDV should be considered in countries with high dengue cases and currently it is not prequalified [1, 12]. Early detection, prompt management and appropriate fluid management are known to reduce dengue mortality.

CPGs have been developed by professional organization for half of century; it was founded on studies during the 1980s related to the geographic variations in clinical practice and appropriateness of care. CPGs are meant to be guidance for clinical practice, based on the best available evidence at the time of development. The development has evolved from consensus based to evidence-based. Reference was also made to other CPGs on dengue such as WHO first published dengue guideline for diagnosis, treatment and control in 1986 which were evaluated prior to them being used as references.

In Malaysia, a group of multidisciplinary specialty from Ministry of Health Malaysia is responsible to “produce” CPGs for various type disease managements. The CPG has been printed and distributed to all public and private health facilities. Softcopy of the CPG is also available to be downloaded from the MOH and Academy of Medicine portal. In 2003, the first edition of dengue CPG was published in Malaysia, and then in 2008 the second edition of dengue CPG was published. The latest edition of Dengue CPG in Malaysia that available during this study was conducted is Clinical Practice Guidelines on Management of Dengue Infection in Adults (Revised 2nd Edition) 2010 which are revised version of previous CPG on Management of Dengue Infection in Adults (2nd Edition) 2008. Currently the latest version of Dengue CPG is the third edition. The main component being revised is the management of fluid in the revised 2^nd^ edition of dengue CPG.

CPG is standardized document develop to harmonize disease management at all levels of healthcare, be it in primary or secondary setting, public and private. A chapter addressing referral criteria is documented in this CPG. These revised 2^nd^ editions of dengue CPG are applicable to primary care doctors, public health personnel, nurses, assistant medical officers, physicians and critical care providers involved in treating adult patient with dengue fever, dengue haemorrhagic fever or dengue shock syndrome and also other severe dengue. Plus, it is appropriate for both outpatient and inpatient settings. There are eight parts in this dengue CPG which is out patient management, patient at emergency management, hospital referral and admission, intensive care management, disease monitoring, fluid management, bleeding management and discharge criteria. The main component in each part is history, assessment of warning sign, physical examination, and assessment of haemodynamic status, diagnosis, investigation, fluid management and discharge criteria.

Clinical practice guidelines will be effective only if they are perceived to be useful and are actually used in clinical decision making. It is therefore important to ensure that clinicians are aware of the guidelines and they actually utilised it in their clinical practice [13]. For that reason, it is significant to evaluate the awareness, utilization and adherence of the dengue CPG among the clinicians. This may give some information on whether the guidelines affect clinicians’ knowledge and behaviour and is there factors contributing to non-compliance with the guidelines. These might inform in development of more effective CPG and if there are any unnecessary criteria in the guideline that should be revised. The Clinical Practice Guideline (CPG) Management of Dengue Infection in Adults (revised 2^nd^ Edition) has been published and disseminated in 2010 to provide evidence-based guidance in the management of dengue infection in adult patients [14]. However, its awareness and utilisation by the target users which includes primary care doctors, public health personnel, physicians and those who are involved in managing dengue cases remain uncertain. Evidence showed that only a proportion of those who utilise the health system actually receive the recommended processes of medical care [15]. Without evaluating the awareness and utilization of this CPG, the effectiveness and quality of the guideline remain uncertain. Therefore, the aim of this study is to assess awareness of the dengue CPG among doctors in Malaysia and utilisation of this CPG in their practice.

## Methods

### Study population and sampling

A cross-sectional study was conducted among registered doctors at primary care and hospital from Medical and Emergency Department both in public and private health facilities in Malaysia to assess their awareness toward the dengue CPG and the proportion of them utilize this CPG. We exclude all private specialist clinics in this study. This study was registered with National Medical Research Register (NMMRID: 20233) and approved by the University of Malaya Medical Centre Ethical Committee (MEC ID: 201412–902).

A total of 860 validated self-administered questionnaires (CPG Awareness and Utilization Feedback Form) were distributed from January 2014 to November 2014.The sample size was estimated based on approximately 40,000 medical practitioners in Malaysia according to the Malaysian Health Fact 2013 by the Ministry of Health (MOH) with consideration of 40% non-response proportion.

Proportionate multistage random sampling was conducted to ensure the representativeness of the samples. The states were clustered into six regions (Central, North, South, East, Sabah and Sarawak), while health facilities were stratified according to hospital and clinics. Estimated numbers of health facilities were based on Malaysian Health Facts 2013. Sampling units which is the medical doctors were randomly selected based on desired sample size per department. A total of 550 clinics (public and private), 65 public hospitals (out of 140 for the whole country), and 25 private hospitals (out of 117 for the whole country) were identified and included in this study (See S1 Fig).

### Questionnaire

The CPG Awareness and Utilization Feedback Form were validated in a pilot study among public doctors in health clinics. The questionnaire comprised of 18 questions from 6 sections: 1) personal details (eg: age, gender, designation, length of service, type of healthcare facility and department) 2) awareness of CPG management of dengue infection in adults 3) training attended 4) utilization of CPG 5) factor associated with utilization, and 6) suggestion to improve utilization of CPG.

### Data collection

The questionnaire was distributed and collected by well trained personnel, which were sent via email and fax. For those who did not return the questionnaire, they were reminded through emails, phone calls, fax and visit if applicable. Their status was coded as non-response after fail to get any feedback after 3 times of reminders.

### Data analysis

Data analysis was performed using IBM SPSS Statistics Version 22. Descriptive statistics were reported. Since the sampling design of this study using the multistage random sampling, complex sample data analysis adjusted for total weights of the sampling design were done. The weights for hospitals and clinics were calculated by dividing total health facility within region with selected health facility within sample frame. Whereas the weights for the health personnel was obtained by total health personnel in the health facility with those that participated in the study. Results were compared between weighted and unweighted and since the results were comparable, therefore we decided to present the unweighted results in this report. Population estimates were presented as prevalence rates.

## Results

### Socio-demographic

**Fig 1:**
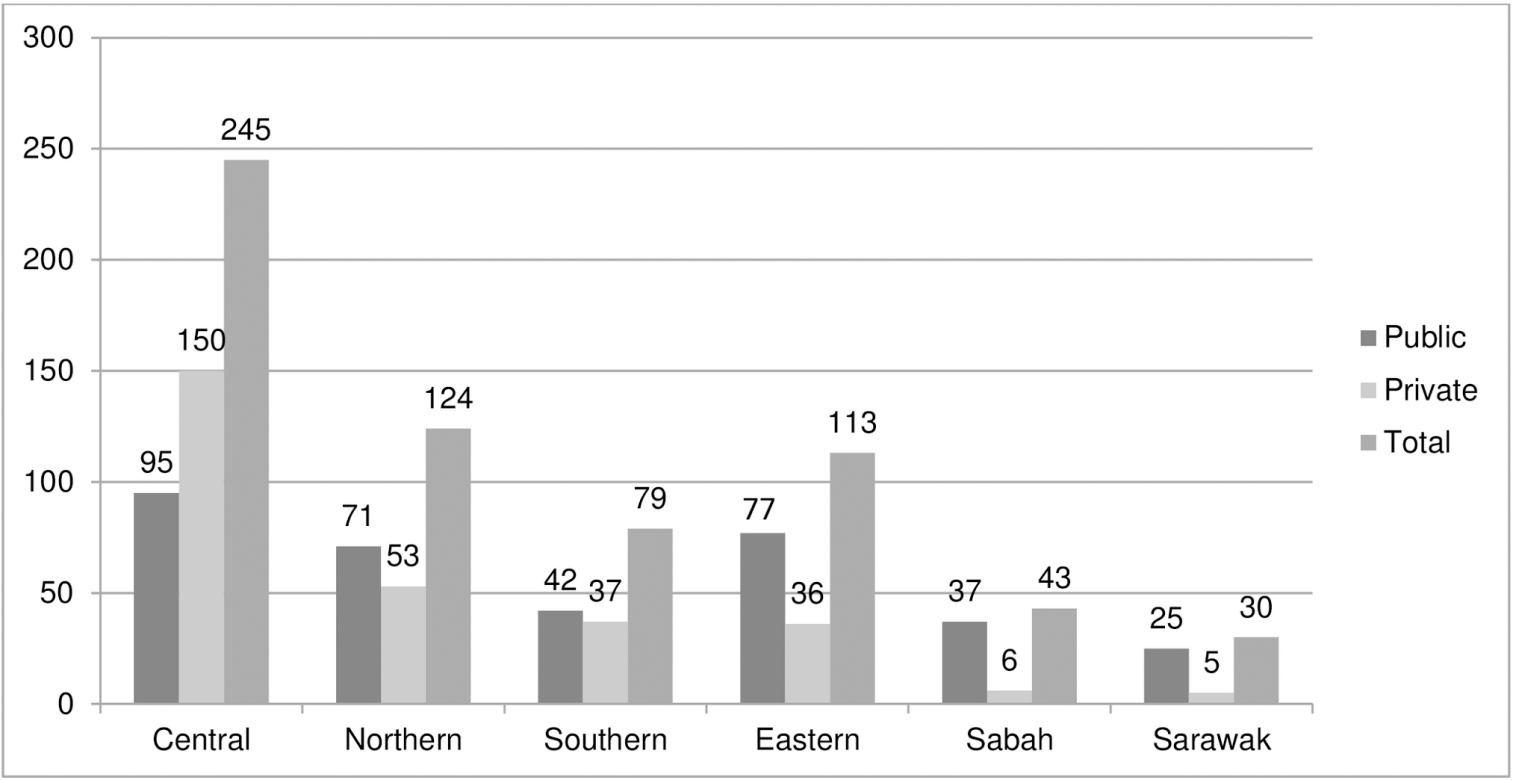
Distribution of respondent by region.

Out of 860 doctors invited to participate in the study, 634 (74%) doctors completed the questionnaire. However, some of the information were not filled by the doctors thus, all percentages were calculated amongst participants with available information only. Response rate were 84% for public hospitals, 82% for private hospitals, 70% for public clinics, and 64% for private clinics (Table 1).Most of respondent from public facilities (95 or 27%) were from Central Region, 71 (21%) from Northern Region, 42 (12%) from Southern Region, 77 (22%) from Eastern Region, 37 (11%) from Sabah and 25 (7%) from Sarawak. From the 287 respondents of private facilities, 150 (52%) were from Central Region, 53 (19%) from Northern Region, 37 (13%) from Southern Region, 36 (13%) from Eastern Region, 6 (2%) from Sabah and 5 (1%) from Sarawak (Fig 1).

**Table 1:** General distribution of respondents comparing public and private health facility.

Variables	Public		Private		Total
	Hospital	Clinic	Hospital	Clinic	
Target sample	260	156	50	394	860
Collected sample	219	128	35	252	634
Proportion of collected sample (%)	84	82	70	64	74

Most (187 or 54%) of respondents from public facilities were below 30 years old, whereas most (156 or 56%) of respondents from private facilities were over 50years old (Table 2). Majority (211 or 61.5%) of respondents from public facilities were female, however only 73 or 26% of respondents from private facilities were female (Table 2).

**Table 2:** Characteristics of respondents by type of health facilities.

Characteristics	Public n (%)	Private n (%)	All n (%)	*P*-Value
**Age group**	n = 343	n = 278	n = 621	<0.0001^a^
< 30 years	187 (54)	4 (1)	191 (31)	
31–40 years	117 (34)	32 (12)	149 (24)	
41–50 years	37 (11)	86 (31)	123 (20)	
> 51 years	2 (1)	156 (56)	158 (25)	
**Gender**	n = 343	n = 286	n = 629	<0.0001^b^
Male	132 (38.5)	213 (74)	345 (55)	
Female	211 (61.5)	73 (26)	284 (45)	
**Personnel**	n = 342	n = 272	n = 614	<0.0001^a^
HO	34 (10)	1 (0.5)	35 (6)	
MO	246 (72)	237 (87)	483 (78)	
Specialist	62 (18)	34 (12.5)	96 (16)	
**Length of service (years)**	n = 347	n = 287	n = 634	<0.0001^c^
Mean (sd)	6.40 (5.38)	23.85 (9.86)	13.98 (11.55)	
Median (IQR: 25th, 75th)	4 (3,9)	24 (16, 30)	10 (4,22)	
Range (min, max)	29 (1, 30)	49 (2, 51)	50 (1, 51)	
**Department**	n = 341	n = 282	n = 623	<0.0001^b^
Medical Department	112 (33)	35 (12)	147 (24)	
Emergency Department	100 (29)	19 (7)	119 (19)	
Clinic (Public & Private)	129 (38)	228 (81)	357 (57)	

^a^*p* values were calculate using the Fisher’s exact test.

^b^*p* values were calculated using the Pearson Chi square test.

^c^*p* value was calculated using the independent sample ttest.

Out of 347 respondents from public hospital 34 (10%) were House Officer (HO), 246 (72%) were Medical Officer (MO) and another 62 (18%) were Specialist. Majority of respondents from private facilities were also among Medical Officer (237 or 87%), followed by Specialist (34 or 12.5%) and one House Officer (Table 2).The overall mean of length of service was 14 years with 6.4 years for public hospital and 24 years for private hospital. The overall maximum length of service was 51 years and minimum of 1 year service (Table 2).

From the 634 respondents, 147 (24%) were from Medical Department with 112 and 35 from public and private hospital respectively, 119 (19%) were from Emergency Department with 100 and 19 from public and private hospital respectively, and another 357 (57%) were from public and private clinic with 129 and 228 respectively (Table 2).

### Awareness of the Dengue CPG

Majority (585 or 93%) of the respondents were aware of the Dengue CPG. A higher proportion of respondents from public facilities (99%) were aware of the CPG compared to those in private facilities (84%) (Table 3). Out of the 345 respondents from public facilities that are aware of the Dengue CPG, 217 were from hospital and 128 were from clinic whereas out of 240respondents from the private facilities, 35 were from hospital and 205 were from private clinic (Table 3).

**Table 3:** Dengue CPG awareness distribution of the respondent comparing public and private facility.

	Public		Private		Total n = 631
	Hospital n = 218 (%)	Clinic n = 128 (%)	Hospital n = 35 (%)	Clinic n = 250 (%)	
Aware	217 (99.5)	128(100)	35 (100)	205 (82)	585 (93)
Not Aware	1(0.5)	0	0	45 (18)	46 (7)

Among the respondents from public facilities, 33/34 (97%) of HO, 245/246 (99%) of MO and all 62 Specialists were aware of the CPG. Whereas among the respondents from private facilities, one (100%) of HO, 198/235 (84%) of MO and 32/34 (94%) of Specialists were aware of the CPG (Table 4).

**Table 4:** Dengue CPG awareness distribution of the respondent comparing designation in public and private facility.

	HO n (%)	MO n (%)	Specialist n (%)	Total	*p* value
**Public**	n = 34	n = 246	n = 62	n = 342	0.100^a^
Aware	33 (97)	245 (99)	62 (100)	340 (99)	
Not aware	1(3)	0	0	1 (1)	
**Private**	n = 1	n = 235	n = 34	n = 270	0.307^a^
Aware	1(100)	198 (84)	32 (94)	231 (86)	
Not aware	0	37 (16)	2 (6)	39 (14)	

^a^*p* value was calculated using the Fisher’s exact test.

For the verification of the awareness of the Dengue CPG question, 548 (94%) of the aware respondent answered primary care doctors is the target user, 425 (74%) answered public health personal is the target user, the 343 (59%) for paramedic and 455 (78%) for physician. There was also some incorrect answer where 70 (12%) answered pharmacist and 29 (5%) answered dietician as the target user of the Dengue CPG (Table 5). From the other Dengue CPG awareness verification part, around 308 were using revised second edition of Dengue CPG, 122 were using second edition, 37 were using revised first and 49 were using first edition of the Dengue CPG. Another 60 respondents claimed that they were using the Third edition of the Dengue CPG (Table 5). This substantial percentages of awareness reflect true awareness among respondents.

**Table 5:** Verification of awareness of Dengue CPG among aware respondent.

	Public n (%) n = 345	Private n (%) n = 240	Total n (%) n = 585
**Target users of the Dengue CPG?**			
Primary care doctors	324 (94)	224 (93)	548 (94)
Public health personnel	268 (78)	157 (65)	425 (74)
Paramedics	240 (70)	103 (43)	343 (59)
Physicians	294 (85)	161 (67)	455 (78)
Pharmacists	50 (15)	20 (8)	70 (12)
Dieticians	19 (6)	10 (4)	29 (5)
**Which edition of CPG you use?**			
First	14 (4)	35 (15)	49 (8)
Revised First	21 (6)	16 (7)	37 (6)
Second	60 (17)	62 (26)	122 (21)
Revised Second	203(59)	105 (44)	308 (53)
Third	48 (14)	12 (5)	60(10)

### Utilization of the Dengue CPG

From the 585 respondents that were aware of the Dengue CPG, 544 (93%) claimed that they utilised the Dengue CPG (Table 6). Out of the 345 respondents that were aware from the public facilities, 338 (98%) claimed utilised the Dengue CPG. In private facilities, among those who were aware of the CPG, 86% (206/240) of them claimed utilised the CPG(Table 6).Detail of the characteristic of respondent who are aware and utilizing the Dengue CPG were shown in Table 6. Most (30 or 83%) of respondents who were not utilising the Dengue CPG were among private doctors in clinic.

**Table 6:** Characteristic of Dengue CPG utilization among aware respondent.

Characteristics	Public n (%)	Private n (%)	All n (%)
**Health facilities**			
Hospital	213 (98)	33 (94)	246 (98)
Clinic	125 (98)	173 (84)	298 (89)
**Personnel**			
HO	32 (97)	1 (100)	33 (97)
MO	241 (98)	167 (84)	408 (92)
Specialist	61 (98)	30 (94)	91 (97)

**Fig 2:**
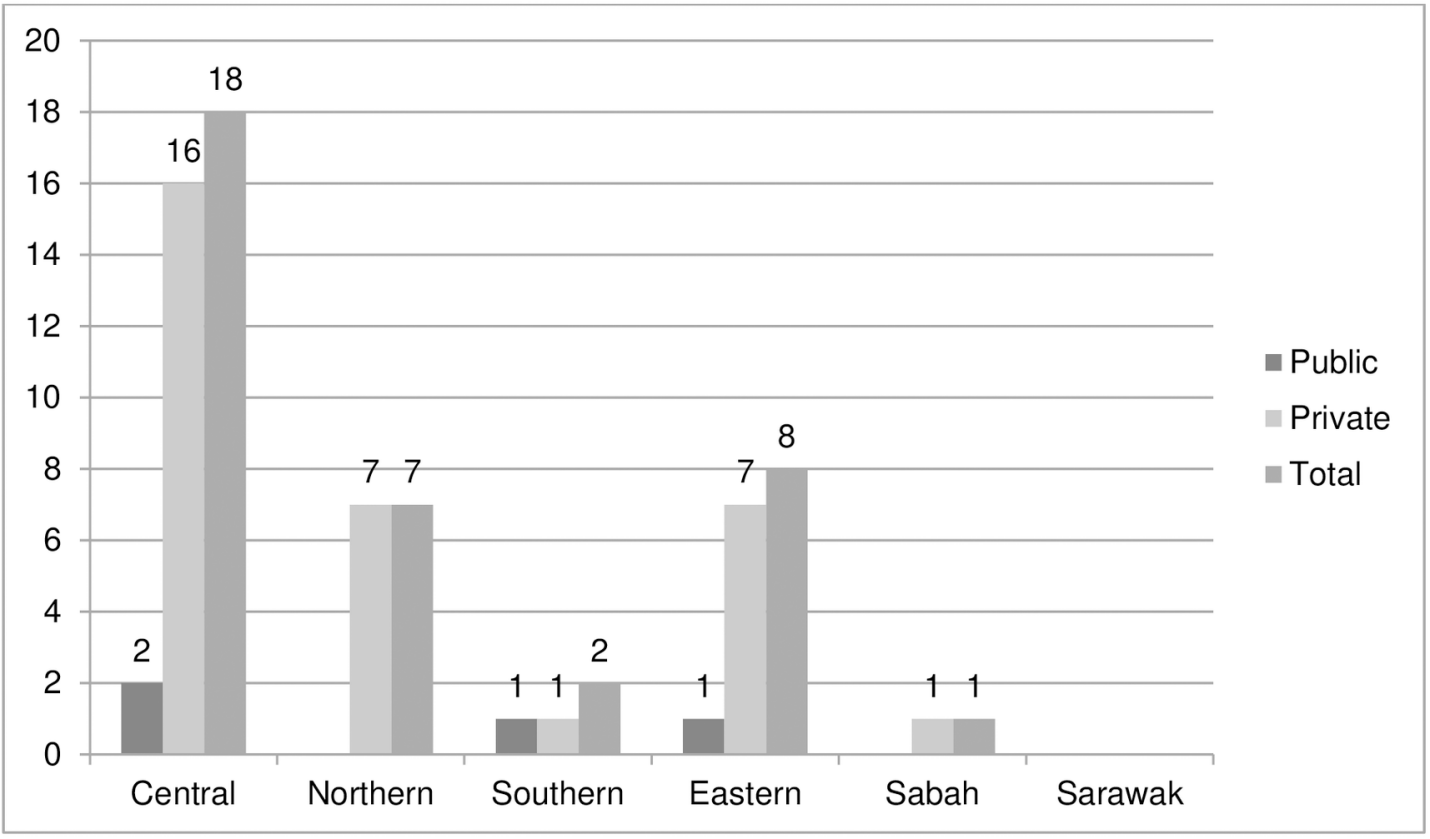
Distribution of respondent who not utilizing Dengue CPG by state.

Fig 2 shows the regional distribution of respondents who did not utilise the CPG. Respondents from Central and Eastern regions show higher percentage (7%) of not utilising the CPG compared to other region (6% in Northern region, 3% in Southern region, 2% in Sabah and none in Sarawak).

Most of the respondent that are utilizing the Dengue CPG stated the reason of using CPG were to assist in decision making in clinical practice, to increase understanding of dengue management and as a reference material. In addition, some of them stated that the reason for utilisation were for the teaching purposes, research purposes and for policy development. Most of the respondent who were not utilizing the Dengue CPG stated that the reason for not utilizing it were lack of resources, lack of time and they already know how to manage dengue patients. In addition, some stated that the reason were the guidelines not accessible and too complicated.

### Preferred form of Dengue CPG for daily practice

From the questionnaire, a preferred form of Dengue CPG for daily practice were asked and 375 (59%) stated quick reference (pocket version) were preferred, followed by 135 (21%) preferred flowchart, and 106 (17%) preferred full CPG version. Mobile application was voted by 293 (46%) of respondents as the most preferred mode of accessing CPG, followed by downloading the CPG from MOH website (259 or 41%). Among those who were aware of the Dengue CPG, only 40% (233/585) ever attended training on the CPG. Nonetheless, majority of respondents (530 or 84%) stated that they would use the Dengue CPG if training is provided.

## Discussion

This is the first study in Malaysia that explores the awareness and utilisation of Dengue CPG among doctors in both public and private facilities. A total of 634 doctors responded to this study, with 345 of them were men and 248 were women. The majority of respondent from the public hospital were younger compared to the private facilities which majority of them age more than 51 years old. This is probably because junior doctors need to serve the government once completed their training. In addition, private hospital created high demand for much senior or specialised doctors with much higher salary [16].

This study showed that most of the doctors were aware of the Dengue CPG. However, the awareness among doctors in private facilities was lower compared to the doctors in public facilities. There are likely to be several reasons for this finding. First, majority of respondents from public facilities were among younger age group compared to those in private facilities. Therefore, they received training from much recent resources and most updated information. Second, respondents from public facilities also have shorter year of services. Junior doctors still have significant knowledge gaps relating to managing their patient [17], and they were more likely to look for guidance compared to those with much longer year of services whom already comfortable in managing their patient based on their experiences. Finally, public facilities usually will conduct training to their staff when receiving new guideline to ensure the awareness and implementation of any new guideline.

Most the respondent that was aware of the dengue CPG claim that they were utilizing the dengue CPG in their dengue management. Utilisations of the Dengue CPG were found to be high among doctors in Malaysia especially in the public facilities. The reasons of using the CPG were mainly, for assisting decision making in clinical practice and as a referral material. However, only about half of them (53%) were using the latest guideline. However, lower utilisations of Dengue CPG among doctors practicing in private clinics were found, especially among the medical officers.

This finding suggests that doctors may not utilise the CPG despite their awareness of the guideline. Doctors may disagree with the recommendation as stated by one of the respondent in this study. Disagreement with the guideline could be due to personal opinion, low quality evidence, transferability/applicability of evidence, or consideration of patient values and preferences [18]. Recommendations in the guideline also may be impractical in some clinical settings because of limitations in consulting time, lack of resources, or lack of attention to the logistics of implementation.

### Strengths and limitations

The limitation of this study mainly was the use of self-reported measure of awareness and utilisation of the CPG instead of objective measurement. On the other hand, this study has the strength of having representative sample from both public and private facilities throughout the country with good response rate. This was a first study in Malaysia that evaluate the Dengue CPG awareness and utilization among doctor and it would provide useful information to the users including health provider as well as programme managers, hospital administrator and relevant policy makers at all level of care.

### Recommendation

Although most of the doctors in Malaysia were aware and did utilize the dengue CPG, there are a proportion of doctors that were not. Some of the suggestions to enhance the level of awareness were to create a link between CPG usage and continuing professional development (CPD), to enhance publication in mass media to reach and widen target users more efficiently. Continuous training could also be conducted as poor training on the guideline can affect the understanding and utilization [5]. Furthermore, encouragement from the department colleague could improve the utilization of the CPG.

The mode of access also plays role in determining the awareness and utilization of doctors to the guidelines [5]. The length and volume of guidelines could be one of the barriers for utilising the CPG doctors may do not have adequate time to read the full details of all guidance. This study found that most of the respondent preferred guideline in a more digestible format, the quick reference with algorithm format, and prefer mobile application as the mode of access.

## Conclusion

In conclusion, we achieved a substantial awareness and utilization level among the doctors in Malaysia. However, utilisation of the CPG among doctors in private clinic still poor. Further research is needed to identify strategies to improve the utilisation of the guideline especially among doctors in private clinic.

## Supporting information

S1 Fig (PDF)
