# Supplementary material for: Evaluation of awareness & utilisation of clinical practise guideline for management of adult Dengue infection among Malaysia doctors
Source: PLoS One. 2017 May 31;12(5):e0178137. doi: 10.1371/journal.pone.0178137 (PMC5451025; doi:10.1371/journal.pone.0178137)
Supplement: S1 Fig — (PDF) [file pone.0178137.s001.pdf]

Region Central (289)

- Hospital (96)
  - Public (80.4)
    - Emergency (48.2)
      - State (24)
      - Specialist (24)
    - Medical (32.2)
      - State (16)
      - Specialist (16)
  - Private (16)
    - Emergency (8)
    - Medical (8)
- Clinic (193)
  - Public (22)
  - Private (171)

Region Northen (177)

- Hospital (60)
  - Public (50)
    - Emergency (30)
      - State (15)
      - Specialist (15)
    - Medical (20)
      - State (10)
      - Specialist (10)
  - Private (10)
    - Emergency (5)
    - Medical (5)
- Clinic (117)
  - Public (31)
  - Private (86)

Region Southern (117)

- Hospital (32)
  - Public (20)
    - Emergency (10)
      - State (5)
      - Specialist (5)
    - Medical (10)
      - State (5)
      - Specialist (5)
  - Private (12)
    - Emergency (6)
    - Medical (6)
- Clinic (85)
  - Public (22)
  - Private (63)

Region Eastern (110)

- Hospital (44)
  - Public (40)
    - Emergency (24)
      - State (12)
      - Specialist (12)
    - Medical (16)
      - State (8)
      - Specialist (8)
  - Private (4)
    - Emergency (2)
    - Medical (2)
- Clinic (66)
  - Public (32)
  - Private (34)

Region Sarawak (95)

- Hospital (40)
  - Public (34)
    - Emergency (20)
      - State (10)
      - Specialist (10)
    - Medical (14)
      - State (7)
      - Specialist (7)
  - Private (6)
    - Emergency (3)
    - Medical (3)
- Clinic (55)
  - Public (35)
  - Private (20)

Region Sabah (72)

- Hospital (38)
  - Public (36)
    - Emergency (20)
      - State (10)
      - Specialist (10)
    - Medical (14)
      - State (7)
      - Specialist (7)
  - Private (2)
    - Emergency (1)
    - Medical (1)
- Clinic (34)
  - Public (14)
  - Private (20)

860 **Desired sample size**
